# Clinical impact of real‐time androgen receptor alteration monitoring on metastatic castration‐resistant prostate cancer treatment in real‐world settings

**DOI:** 10.1002/ijc.70019

**Published:** 2025-06-25

**Authors:** Regina Stitz, Franz Stoiber, Renè Silye, Elisabeth Rebhan, Michael Dunzinger, Franz Pühringer, Ellen Heitzer, Cornelia Hauser‐Kronberger

**Affiliations:** ^1^ Department of Pathology Salzkammergutklinikum Vöcklabruck Vöcklabruck Austria; ^2^ Doctoral Program Medical Science Paracelsus Medical University Salzburg Salzburg Austria; ^3^ Department of Pathology Klinikum Wels‐Grieskirchen Wels Austria; ^4^ Department of Urology Medicine Salzkammergutklinikum Vöcklabruck Vöcklabruck Austria; ^5^ Institute of Human Genetics, Diagnostic & Research Center for Molecular BioMedicine Medical University of Graz Graz Austria; ^6^ Christian Doppler Laboratory for Liquid Biopsies for Early Detection of Cancer Medical University of Graz Graz Austria; ^7^ Department of Anatomy and Cell Biology Paracelsus Medical University Salzburg Salzburg Austria

**Keywords:** androgen receptor, AR‐V7, liquid biopsy, monitoring, prostate cancer

## Abstract

Androgen receptor (*AR*) alterations contribute to resistance against androgen receptor signaling inhibitors (ARSi) in metastatic castration‐resistant prostate cancer (mCRPC). This study evaluated *AR* alteration monitoring via liquid biopsy in routine clinical practice. To this end, we enrolled 39 mCRPC patients in a real‐world clinical setting and monitored disease progression with progression‐free survival (PFS) and overall survival (OS) analyzed in relation to *AR* status. *AR* alterations were detected in 8 of 39 patients (20.5%) at baseline, with five additional cases emerging during progression (total 33.3%). AR‐V7 was identified in 12.8%, and *AR* amplification and/or hotspot mutations in 20.5%. Patients with *AR* alterations had significantly lower PSA response rates to ARSi (37.5% vs. 80.7%; *p* = 0.0276). All *AR* alteration‐positive patients experienced disease progression, compared to 34.6% of *AR*‐negative cases. PFS was significantly shorter in *AR* alteration‐positive patients (11 vs. 52 months; *p* = 0.001), while OS showed a non‐significant trend toward shorter survival (41 vs. 74 months; *p* = 0.0619). Univariate analysis confirmed *AR* alterations as an independent predictor of PFS (*p* = 0.0035). This real‐world study demonstrates that *AR* monitoring via liquid biopsy predicts treatment response and progression in mCRPC. Continuous monitoring is essential, as *AR* alterations emerge over time. Patients with *AR* alterations have poorer ARSi responses and shorter PFS, emphasizing the need for adaptive treatment strategies in real‐world clinical practice.

## What's New?

Androgen receptor (*AR*) alterations contribute to treatment resistance against androgen receptor signaling inhibitors in metastatic castration‐resistant prostate cancer. This real‐world study demonstrates that *AR* monitoring via liquid biopsy predicts treatment response and progression in metastatic castration‐resistant prostate cancer. It highlights the dynamic nature of *AR* alterations, their role in resistance to *AR* inhibitors, and their association with poor outcomes. Patients with *AR* aberrations may benefit more from chemotherapy, emphasizing the need for predictive biomarkers. Liquid biopsy may serve as a non‐invasive, cost‐effective tool for routine real‐time *AR* monitoring, enabling adaptive cancer therapy in metastatic castration‐resistant prostate cancer in clinical practice.

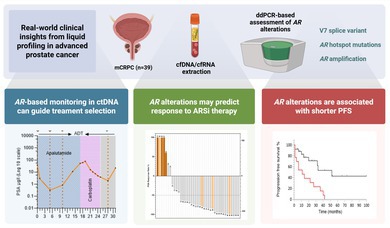

**Abbreviations**
AA: Abiraterone acetate
ADT: Androgen deprivation therapy
APA: Apalutamide
AR: Androgen receptor
ARSi: Androgen receptor signaling inhibitors
AR‐FL: Androgen receptor full‐length transcript
AR‐V7: Androgen receptor splice variant 7
CAB: Cabazitaxel
cfDNA: Cell‐free DNA
CNA: Copy number alteration
CRPC: Castration‐resistant prostate cancer
CT: Computed tomography
DARO: Darolutamide
DNA: Deoxyribonucleic acid
DOC: Docetaxel
ENZ: Enzalutamide
HSPC: Hormone‐sensitive prostate cancer
IV: Intravenous
KLK3: Kallikrein‐related peptidase 3 (PSA gene)
LDH: Lactate dehydrogenase
MA: Massachusetts
mCRPC: Metastatic castration‐resistant prostate cancer
NE: Nebraska
OS: Overall survival
PCR: Polymerase chain reaction
PFS: Progression‐free survival
PSA: Prostate‐specific antigen
PSA50: ≥50% reduction in PSA levels
RECIST: Response evaluation criteria in solid tumors
RLT: Radioligand therapy
RNA: Ribonucleic acid
RPE: Radical prostatectomy
SNV: Single nucleotide variant

## 1 INTRODUCTION

Prostate cancer is the most commonly diagnosed cancer in men and the second leading cause of cancer‐related deaths in the Western population.^1^, ^2^ While patients diagnosed with localized prostate cancer have excellent 5‐year survival rates, the median overall survival (OS) of metastatic prostate cancer is only 3–5 years.^3^ As the androgen receptor (AR) plays an important role in the progression of prostate cancer, mediating the effects of androgens on cellular proliferation and survival,^4^ a systemic lowering of androgen levels through androgen deprivation therapy (ADT) is the first‐line treatment. While ADT is initially effective in controlling hormone‐sensitive prostate cancer (HSPC), most patients eventually develop resistance, leading to castration‐resistant prostate cancer (mCRPC), which further declines OS to 1–3 years, depending on prior treatments and biomarker status. For patients with mCRPC, the next therapeutic step after ADT failure is the use of androgen receptor signaling inhibitors (ARSi), which more effectively block AR activity.^5^ These therapies have significantly improved survival in mCRPC,^6^, ^7^ yet resistance to ARSi remains a major challenge, limiting long‐term effectiveness.

Recent studies have demonstrated that alterations in the *AR*—such as amplifications, splice variants (e.g., AR‐V7), and point mutations—are strongly associated with resistance to ARSi.^8^, ^9^, ^10^, ^11^, ^12^ Among these, AR‐V7 has emerged as a particularly strong predictive biomarker for ARSi resistance, while *AR* amplifications and point mutations‐especially in the ligand‐binding domain—may contribute to variable levels of resistance and are often associated with poorer clinical outcomes. Some point mutations within this domain are known to mediate resistance by converting antagonists like enzalutamide into partial agonists, while others broaden the receptor's ligand specificity, allowing activation by alternative steroids such as progesterone or by glucocorticoids like prednisone.^8^, ^9^, ^10^, ^12^, ^13^, ^14^, ^15^, ^16^

Identifying these alterations may allow for better treatment selection and optimization of therapeutic sequences. Previously, we have developed and validated five multiplex droplet digital PCR (ddPCR) assays for detecting *AR* amplification, AR‐V7, KLK3 expression, and *AR* point mutations from circulating nucleic acids derived from a liquid biopsy. Unlike traditional tissue biopsies, liquid biopsies can provide a comprehensive snapshot of tumor heterogeneity and dynamics over the course of treatment. Several studies have demonstrated the feasibility of detecting *AR* alterations in liquid biopsy samples and have correlated these alterations with clinical outcomes in mCRPC patients.^13^, ^17^, ^18^, ^19^

Our assays demonstrated excellent analytical sensitivity and specificity, confirming their potential utility in clinical practice. Consistent with other studies, our findings indicated that *AR* alterations were significantly more prevalent in patients with CRPC compared to those in the hormone‐sensitive stage.^13^, ^17^, ^18^, ^19^ Building on our previous findings, this study evaluates the clinical utility of *AR* alteration monitoring in a real‐world cohort of mCRPC patients using liquid biopsy. Unlike controlled clinical trials, this observational study provides real‐world evidence from patients receiving standard‐of‐care treatments, reflecting actual clinical practice. By analyzing cfDNA and cfRNA from 39 patients, we assessed the prevalence and evolution of *AR* alterations and their impact on treatment response and survival outcomes. This research contributes to the growing body of evidence supporting the integration of liquid biopsy into routine patient management, enabling personalized and adaptive treatment strategies for advanced prostate cancer in real‐world settings.

## 2 MATERIALS AND METHODS

### 2.1 Patients and Samples

A total of 103 plasma samples were analyzed from 39 men diagnosed with mCRPC, who were at various stages of treatment at the Salzkammergutklinikum between July 2022 and June 2024. All patients had a histologically confirmed diagnosis of prostate adenocarcinoma and were classified as having CRPC according to the guidelines of the European Association of Urology. Blood samples were collected from patients before therapy initiation and/or during treatment as part of routine clinical care, with collection timing determined by the attending physician's discretion to gather additional information for treatment decisions. No specific study protocol or standardized guidelines dictated the timing of sample collection. Up to four follow‐up assessments were conducted to monitor *AR* alteration status throughout the disease course, with treatment strategies adjusted based on *AR* alteration findings.

### 2.2 cfDNA and cfRNA extraction from plasma and ddPCR analysis

A minimum of 20 mL peripheral blood was collected from each patient into three cfRNA blood collection tubes (Streck, Omaha, NE). Plasma was extracted within 72 h using a double spin protocol (1800×*g* for 15 min, 2800×*g* for 15 min) at the Department of Pathology at Salzkammergutklinikum as recommended by the manufacturer.

Total nucleic acids, including cfDNA and cfRNA, were isolated from 10 mL of plasma (2 × 5 mL) using the QIAamp Circulating Nucleic Acid Kit (QIAGEN), according to the manufacturer's instructions, and eluted in 60 μL of elution buffer. Plasma‐derived cfRNA was extracted without adding carrier RNA as previously described.^20^ The total nucleic acid eluates were purified further with the RNeasy MinElute Cleanup Kit (QIAGEN) and eluted in 14 μL of RNase‐free water. Of the 14 μL eluted cfRNA, 11 μL was reverse transcribed using a modified reverse transcription protocol with SuperScript IV reverse transcriptase (Thermo Fisher Scientific, Waltham, MA).

Five multiplex ddPCR assays were used for the detection of 13 *AR* targets including *AR* amplification [copy number alteration (CNA)], AR‐V7, KLK3, and a total of 10 *AR* hotspot mutations in codons 702, 742, 875, 877, and 878. The CNA assay was designed using primers and probes targeting the *AR* gene, along with three validated reference genes: *ARHGEF9*, *NSUN3*, and *AGO1*. For cfRNA gene expression targets—AR‐FL, AR‐V7, and KLK3—HPRT1 served as the endogenous control. Each reaction included no‐template controls and positive controls with known target profiles to ensure assay performance. Due to the design of the hotspot mutation assays, mutations at codons 877 and 878, as well as 702 and 742, cannot be distinguished individually. Consequently, results from these regions are reported as *AR* p.T878A/S‐F877L and *AR* p.L702H‐W742L/C, respectively. The details, development, and performance characteristics of all five assays have been thoroughly described previously.^20^

### 2.3 Statistical analysis

Kaplan–Meier method was used to evaluate time‐to‐event outcomes, including progression‐free survival (PFS) and overall survival (OS). PFS was defined as the interval from the initiation of the current ARSi therapy, or from the start of the most recent ARSi therapy if treatment had already concluded, until documented disease progression or death. OS was measured from the initiation of the first ARSi therapy to the time of death from any cause. Survival differences were compared by the log‐rank test.

Biochemical progression was defined as a ≥25% increase and an absolute rise of ≥2 ng/mL above the nadir, confirmed by a second consecutive measurement at least 3 weeks later. Clinical and/or radiographic progression was defined as either symptomatic progression (worsening symptoms related to the disease), radiologic progression (≥20% increase in the sum of diameters of soft‐tissue target lesions on CT scan, according to RECIST [Response Evaluation Criteria in Solid Tumors]), the appearance of two or more new bone lesions on bone scan, or death—whichever occurred first.

The PSA response rate was calculated according to the Prostate Cancer Clinical Trials Working Group 2 (PCWG2) criteria, which define a PSA response as a decline of ≥50% from baseline, confirmed by a second measurement at least 3 weeks later. In our study, baseline was defined as the initiation of first‐ or second‐line ARSi therapy.

The statistical significance of the difference in PSA response rates between *AR*‐alteration positive and negative groups was assessed using Fisher's exact test, with significance defined as a *p*‐value <0.05. Categorical variables, such as Gleason score at diagnosis, presence of bone, lymph node, and visceral metastases, and type of hormonal treatments, were described by absolute and relative frequencies.

All statistical analyses were performed using GraphPad Prism version 10.4.0 (GraphPad Software, Inc., La Jolla, CA).

## 3 RESULTS

### 3.1 Patient characteristics and timing of blood sampling

The primary objective of this study was to evaluate the clinical utility of *AR* alteration monitoring in routine oncology practice. Between July 2022 and June 2024, we enrolled 39 patients with CRPC with a median survival of 59 months and a median follow‐up time of 52 months (range, 4–100) and monitored their progress through September 2024. A subset of patients (*n* = 19) was previously described in Stitz et al.^20^ Patients' median age was 77 years (range, 60–87); Bone and/or lymph node metastases were observed in all patients (100%), while only one patient (2.6%) had additional visceral metastases. Patient characteristics are summarized in Table 1. Using real‐world clinical data, we assessed PFS and OS in relation to *AR* status. A secondary objective was to investigate the detectability and temporal dynamics of *AR* alterations in response to ARSi and chemotherapy. However, due to the limited number of CRPC patients within the cohort, *AR* testing prior to the initiation of the first ARSi treatment was only feasible in a subset of patients (*n* = 6). The majority of patients had already initiated ARSi therapy at the time of their first *AR* assessment, thereby influencing the ability to evaluate *AR* alterations in an ARSi treatment‐naïve context.

**TABLE 1:** Study population characteristics presented as mean (range) or number (percentage).

	Total (*n* = 39)
Patients (CRPC)	39
Mean age (years)	77 (60–87)
Gleason score (%)	
≤7	10 (25.6)
>7	26 (66.7)
N.A.	3 (7.7)
Metastatic status (%)	
Bone metastases	32 (82.2)
Lymph metastases	23 (59.0)
Visceral metastases	1 (2.6)
Definitive therapy (%)	
Radical Prostatectomy	10 (25.6)
Radiotherapy	28 (71.8)
Both	6 (15.4)
None	7 (17.9)
Systemic therapy (prior and/or current use)	
ADT	38 (97.4)
CYP17A inhibitor (Orteronel)	1 (2.6)
Chemotherapy	14 (35.9)
None	0 (0.0)
Novel antiandrogens (prior or current use)	
Abiraterone (AA) only	17 (43.6)
Enzalutamide (ENZ) only	8 (20.6)
Apalutamide (APA) only	3 (7.7)
Darolutamide (DARO) only	1 (2.6)
APA followed by ENZA and/or AA	5 (12.8)
ENZA followed by AA	3 (7.7)
AA followed by APA	1 (2.6)
DARO followed by APA and ENZA	1 (2.6)

Abbreviations: AA, abiraterone; ADT, androgen deprivation therapy; APA, apalutamide; DARO, darolutamide; ENZ, enzalutamide; N.A., not available.

At the time of the first blood draw, 10 patients had not yet started their first or second line ARSi therapy. A total of 23 patients were receiving ongoing ARSi treatment (abiraterone, *n* = 7; enzalutamide, *n* = 8; apalutamide, *n* = 7; darolutamide, *n* = 1), while six patients received either non‐ARSi therapies (e.g., chemotherapy, radioligand therapy) or no systemic treatment.

**FIGURE 1:**
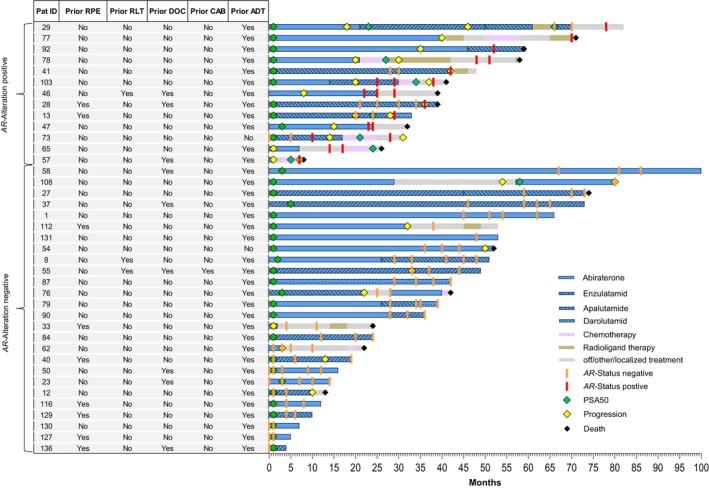
Swimmer plot by ARSi treatment, timing and results of *AR* status determination, progression and response. Each lane is color‐coded based on whether the patient received ARSi therapy (light blue with different patterns), chemotherapy (pink), radioligand therapy (olive) or no systemic therapy (gray). Orange and red vertical bars indicate the timing and result of the measurement (*AR* status negative vs. *AR* status positive). Diamonds indicate PSA response >50% (green), PSA progression (yellow) or death (black). ADT, Androgen Deprivation Therapy; CAB, cabazitaxel; DOC, docetaxel; RPE, radical prostatectomy; RLT, radioligand therapy.

Within the entire cohort, 22 exhibited biochemical, clinical, and/or radiographic progression, while 17 patients did not experience disease progression during the study period. Of those who progressed, 15 patients underwent *AR* testing at or after their first progression event, while seven patients were tested prior to disease progression. The treatment regimen, progression status, PSA response (defined as a ≥50% reduction in PSA levels, PSA50), along with the timing and results of *AR*‐status assessment are presented in Figure 1.

### 3.2 Plasma *AR* status and PSA response rate

**FIGURE 2:**
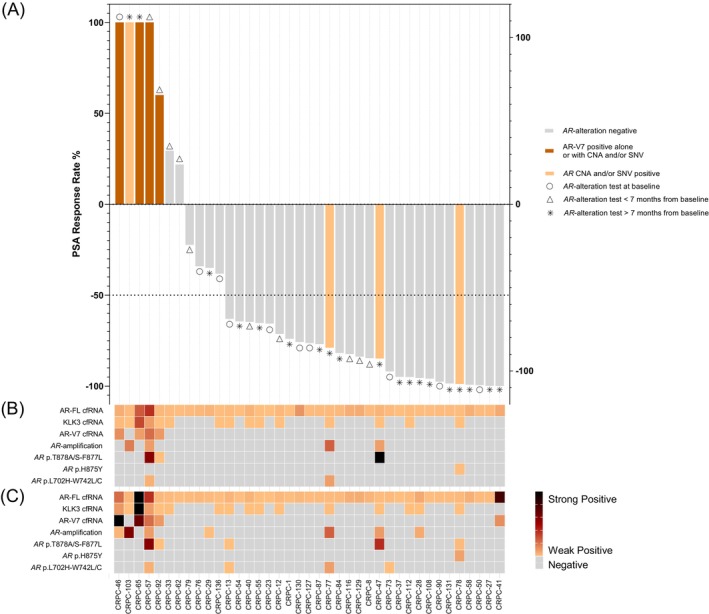
Waterfall plot of best prostate‐specific antigen (PSA) responses by *AR* alteration status and heatmaps of detected *AR* alterations at baseline and at last follow‐up. (A) Waterfall plot showing PSA change (%) on the Y‐axis and individual patients on the X‐axis. Asterisks (*), triangles (∆), and circles (○) indicate the timepoint of *AR* testing: At baseline (*n* = 10), <7 months after ARSi initiation (*n* = 10), and >7 months after ARSi initiation (*n* = 19), respectively. Bar colors represent *AR* alteration status: Gray for *AR* alteration‐negative, dark orange for AR‐V7 positive alone or in combination with *AR* amplification (CNA) and/or *AR* hotspot mutations (SNV), and light orange for *AR* CNA and/or *AR* SNV positive. (B, C) Heatmaps display cfRNA markers including AR‐FL, KLK3, AR‐V7, as well as *AR* amplification and hotspot mutations at codons 702, 742, 875, 877, and 878. The orange to black gradient indicates expression or mutation intensity; gray indicates absence of the target. (B) *AR* alterations at the first blood draw, (C) *AR* alterations at the last follow‐up. *AR*, androgen receptor; cfRNA, cell‐free RNA; *AR* CNA, *AR* amplification; *AR* SNV, *AR* hotspot mutations; AR‐FL, androgen receptor full‐length; KLK3, kallikrein‐related peptidase 3; AR‐V7, androgen receptor splice variant 7.

**FIGURE 3:**
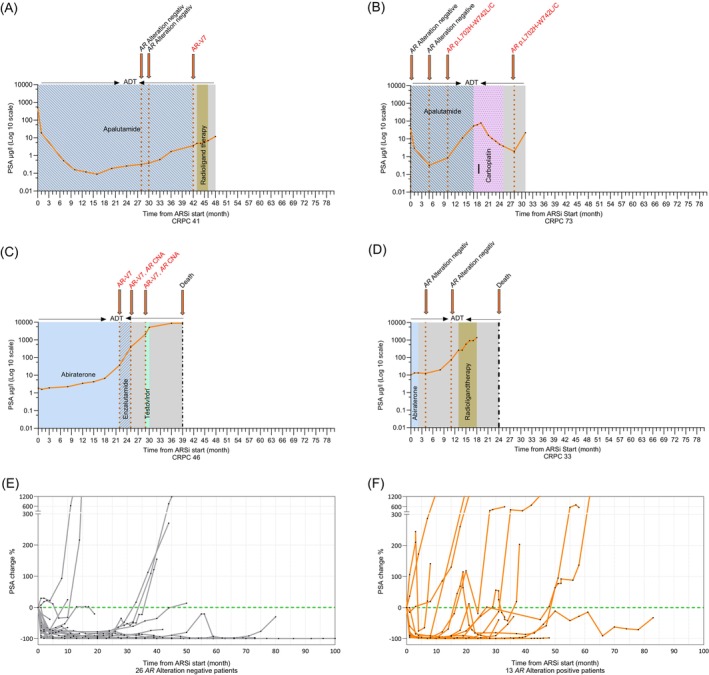
A representative panel of individual PSA progression in *AR* alteration‐positive and ‐negative patients, along with a comparison of PSA change rates between the two groups. Panels A–D display individual PSA trajectories on a log10 scale, spanning from the initiation of the first ARSi therapy to either the end of the study observation period or the patient's death. Each panel indicates the *AR* status and highlights key treatment interventions. Treatments are represented as ARSi therapy (light blue with various patterns), chemotherapy (pink), radioligand therapy (olive), or no systemic therapy (gray). PSA trajectories for all patients are shown in Figures S1, S2 and S3. Panels E and F provide a grouped comparison of PSA change rates (%) between 26 *AR* alteration‐negative and 13 *AR* alteration‐positive patients, with the PSA change rate represented on the y‐axis. ADT, androgen deprivation therapy; *AR*, androgen receptor; ARSi, androgen signaling inhibitor therapie; SNVs*, *AR* p.T878A/S‐F877L + *AR* p.L702H‐W742L/C.

Considering only the first blood draw, *AR* alterations were detected in 8 of 39 patients (20.5%) (Figure 2B). During the observation period, five additional patients, who initially had *AR* wild‐type status, subsequently presented detectable *AR* alterations in a follow‐up sample (Figures 3A and S1A–D). Therefore, altogether *AR* alterations were identified in 13 of 39 patients (33.3%) (Figure 2C). Among these, the AR‐V7 splice variant was identified in five patients (12.8%), while *AR* amplification and/or *AR* hotspot mutations were observed in eight patients (20.5%). Furthermore, seven of those patients (54%) harbored more than one *AR* alteration, with five patients exhibiting a combination of AR‐V7, *AR* amplification, and/or hotspot mutations, and two patients displaying multiple *AR* hotspot mutations. In total, we identified 22 *AR* altering events across the 13 patients: five AR‐V7 results, seven *AR* gene amplifications findings, and 10 *AR* hotspot mutation detections (Figure 2C). Moreover, 12 patients (92.3%) also had detectable levels of KLK3, while all tested patients exhibited detectable full‐length AR (AR‐FL) transcript levels confirming the presence of amplifiable and intact cfRNA from prostate‐derived tissue in liquid biopsy samples (Figure 2C).

To assess the predictive value of *AR* alteration screening, we calculated the PSA response rates of all 39 patients according to their *AR* alteration status (Figure 2A). Six patients were receiving either non‐ARSi therapy or no systemic treatment at the time of sample collection; however, all had previously received abiraterone, with three patients demonstrating no therapeutic response to abiraterone.

Among the 39 mCRPC patients analyzed, 28 (71.8%) exhibited a PSA decline of >50% following treatment. However, the PSA response rate to ARSi therapy was significantly lower in patients with *AR* alterations, with only 37.5% (3 of 8 patients) responding, compared to 80.7% (25 of 31 patients) in *AR* alteration‐negative cases (Fisher's exact test, *p* = 0.0276) (Figure 2A). When considering only AR‐V7, the difference in response to treatment is even more significant. None of the patients with AR‐V7 expression (0 of 4) showed a PSA response, whereas 80% of AR‐V7 negative patients (28 of 35) responded to therapy (Fisher's exact test, *p* = 0.004). In contrast, patients with *AR* amplifications or hotspot mutations demonstrated lower response rates compared to negative individuals, although these differences were not statistically significant. Response rates were 50% in patients with *AR* amplification versus 74.3% in those without (*p* = 0.5619), and 60% versus 73.5% in patients with and without *AR* hotspot mutations, respectively (*p* = 0.6088). These findings should be interpreted with caution, as not all patients were tested for *AR* alterations at baseline. Consequently, some patients classified as *AR* alteration‐positive may have acquired these alterations during therapy, particularly those who were tested more than 7 months after treatment initiation (indicated with * in Figure 2), potentially affecting the observed response rates.

### 3.3 Plasma *AR* status and disease progression

All 13 patients (100%) with *AR* alterations experienced tumor progression and demonstrated resistance to ongoing or prior ARSi therapy (Figures 3A–C and S1). In contrast, only 34.6% (9/26) of patients without detected *AR* alterations experienced disease progression while receiving ARSi treatment (Figures 3D and S2). Among the 13 patients with *AR* alterations, five initially presented a biochemical PSA response to ARSi therapy without detectable *AR* alterations during the response phase. However, *AR* alterations emerged subsequently, coinciding with radiographic or biochemical disease progression. Specifically, the detected *AR* alterations included two cases of *AR* amplifications, one patient with AR‐V7 expression, and two patients with hotspot mutations. At the time of *AR* alteration detection, the average PSA level was 4.38 μg/L (range: 1.41–12.3 μg/L) (Figures 3A,B and S1A–E). Notably, in one of these five cases (Figure S1B), an *AR* amplification was detectable prior to the rise in PSA levels, indicating that molecular progression may precede biochemical progression.

Following ARSi failure, six of the 13 *AR* alteration‐positive patients received chemotherapy, all of whom demonstrated improved responses compared to their prior ARSi treatment (Figures 3B and S1E–J). For four of these patients, liquid biopsy samples were available either before or after chemotherapy, while in two cases, samples were collected both before and after treatment. Notably, one patient exhibited a persistent hotspot mutation in the codon 702–742 region, detected both pre‐ and post‐chemotherapy, with only a slight decrease in the number of mutant copies per milliliter of plasma during treatment—from 40 copies/mL before therapy to 35 copies/mL after therapy (Figure 3B). Another patient had an *AR* amplification, with a modest change in copy number level, decreasing from 5.24 before chemotherapy to 3.65 after treatment (Figure S1H). These findings suggest that while chemotherapy can lead to clinical improvement in patients, *AR* alterations, such as mutations or amplifications, may persist despite treatment.

Additionally, three *AR* alteration‐positive patients who initially exhibited therapeutic response or stable disease later developed tumor progression with PSA levels exceeding 20 μg/L, coinciding with the emergence of *AR* alterations. However, liquid biopsy from the earlier response or stable disease phase (PSA levels below 20 μg/L) was unavailable for these patients. Among the three patients, two showed AR‐V7 expression alongside additional *AR* alterations, while one carried an *AR* amplification in combination with a mutation at codons 878–877 (Figures 3C and S1K–M). After approximately 20 months of ARSi therapy, PSA levels rose markedly in both AR‐V7 positive patients, with no response to treatment switch, indicating AR‐V7‐associated resistance.^13^, ^15^ The third patient progressed under abiraterone, likely due to the mutation at codons 878–877, which is known to expand AR ligand specificity and may be activated by abiraterone‐induced progesterone levels^12^, ^21^ (Figures 3C and S1L).

In four cases with AR‐V7 positivity and/or *AR* amplification, longitudinal monitoring revealed dynamic changes in the concentration of AR‐V7 expression and *AR* CNA. Three patients showed rising *AR* CNAs alongside increasing PSA levels, with CN values shifting from 1.23, 1.63, and 1.97 to 1.35, 1.66, and 3.65, respectively (Figure S1K–L,H). One of them also developed AR‐V7 expression, which rose from 7.8 to 49 copies/mL under enzalutamide, indicating complete treatment failure (Figures 3C and S1K). The fourth patient, assessed post‐abiraterone, also showed a strong increase in PSA (304 to 1754 μg/L) and AR‐V7 (5.8 to 33.3 copies/mL; Figure S1G). These molecular alterations were accompanied by parallel upregulation of AR‐FL and KLK3 transcript levels, as illustrated in the heatmaps presented in Figure 2B,C.

Among the 26 *AR* alteration‐negative patients, nine experienced tumor progression. Of these, two exhibited primary resistance to ARSi therapy, showing no PSA decline after treatment initiation, while seven initially responded to ARSi but later developed tumor progression. Unfortunately, additional advanced stage information—such as markers for neuroendocrine differentiation, histopathological data, or genomic sequencing data—were unavailable for these patients, preventing conclusions regarding the mechanisms underlying their primary or secondary resistance to ARSi therapy (Figures 3D and S2). The remaining 17 *AR* alteration‐negative patients did not develop tumor progression during the study observation period and are depicted in Figure S3.

In summary, patients without *AR* alterations tend to have a more stable disease course, with relatively consistent PSA trajectories over time (Figure 3E). In contrast, *AR*‐alteration‐positive patients display more dynamic PSA fluctuations, characterized by both PSA increases and transient declines, indicative of a more heterogeneous treatment response and disease progression pattern (Figure 3F). Our findings further suggest that real‐time *AR* monitoring may detect resistance earlier than PSA alone. While AR‐V7 and *AR* CNA levels often correlate with PSA, *AR* alterations can support therapy decision‐making beyond conventional biomarkers.

### 3.4 Progression‐free survival and overall survival

**FIGURE 4:**
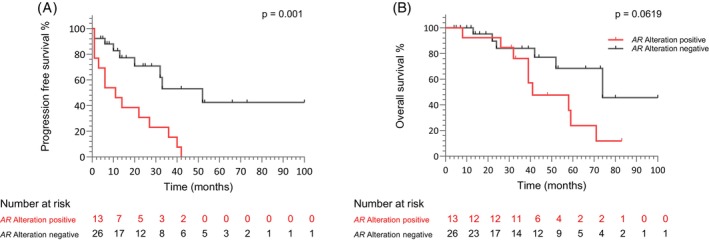
Kaplan–Meier curves depicting (A) progression‐free survival and (B) overall survival stratified by *AR* alteration status (positive vs. negative). The number of patients at risk at each time point for both *AR* alteration‐positive and *AR* alteration‐negative groups is displayed below each plot. *AR*, androgen receptor.

Patients with *AR* alterations exhibited a significantly shorter median PFS of 11 months compared to 52 months in *AR‐*negative patients (*p* = 0.001; Figure 4A). Similarly, the median OS was shorter in patients with *AR* alteration (41 vs. 74 months) compared to those without detected *AR* alterations; however, this difference did not reach statistical significance (*p* = 0.0619; Figure 4B). Due to the limited number of progression and survival events, a stratified analysis based on the different categories of *AR* alterations was not feasible.

To assess the prognostic significance of *AR* status, univariate Cox proportional hazard models were applied to evaluate time‐to‐event outcomes and test for independence from other clinical parameters. Among the variables analyzed, *AR* alterations emerged as an independent predictor for PFS (*p* = 0.0022), alongside anemia, while site score, PSA, and LDH were not statistically significant (Table 2). Due to the limited number of events in each cohort, multivariable models were not performed.

**TABLE 2:** Univariate Cox proportional hazard analysis assessing independence from other clinical parameters.

Progression free survival			Overall survival		
Variable	HR (95% CI)	*p* value	Variable	HR (95% CI)	*p* value
*AR*‐Status	3.992 (1.673 to 10.14)	0.0022	*AR*‐Status	2.618 (0.9368 to 7.864)	0.0699
Hb (>13, <13)	5.817 (2.028 to 19.03)	0.0016	Hb (>13, <13)	5.333 (1.781 to 15.66)	0.0021
Site Score (≤1, >1)	0.9722 (0.3867 to 2.280)	0.9496	Site Score (≤1, >1)	1.803 (0.5658 to 5.628)	0.3035
PSA μg/L	0.9985 (0.9928 to 1.002)	0.4967	PSA μg/L	0.9930 (0.9616 to 1.002)	0.3813
LDH (>250, <250 U/L)	1.965 (0.5471 to 5.673)	0.2443	LDH (>250, <250 U/L)	2.740 (0.7416 to 8.297)	0.0927

Abbreviations: *AR*‐Status, androgen receptor status; Hb (>13, <13), hemoglobin levels greater than 13 g/dL or less than 13 g/dL; HR (95% CI), hazard ratio (95% Confidence Interval); LDH (>250, <250 U/L), lactate dehydrogenase levels greater than 250 units per liter or less than 250 units per liter; PSA μg/L, prostate‐specific antigen concentration as a continuous variable in micrograms per liter; Site Score (≤1, >1), number of metastatic sites, with scores of 1 or less versus more than 1.

## 4 DISCUSSION

This observational study, conducted in a *real‐world clinical setting*, provides valuable insights into the clinical utility of *AR* alteration monitoring in mCRPC patients using liquid biopsy. Unlike controlled clinical trials, which often follow standardized protocols, this study reflects real‐world treatment dynamics, where therapeutic decisions are made based on the evolving clinical status of each patient. The high detection rate of *AR* alterations (33.3%) in this cohort, along with their association with inferior clinical outcomes ‐ such as lower PSA response rates and higher rates of disease progression ‐ supports the potential role of *AR* status assessment in routine clinical practice.

Patients harboring *AR* alterations exhibited poorer therapeutic responses, with a significantly lower PSA response rate to ARSi therapy (37.5%) compared to patients without *AR* alterations (80.7%). Given the distinct functional consequences of AR‐V7 expression, *AR* amplification, and hotspot mutations, we analyzed response rates for each alteration individually.^13^, ^14^, ^15^, ^22^, ^23^, ^24^ AR‐V7 was strongly associated with primary resistance, with no responders observed in this subgroup (0%) while *AR* amplification and *AR* hotspot mutations showed a trend toward reduced efficacy, albeit less distinctly (50% and 60%). These findings are consistent with data reported by Armstrong et al., showing a response rate of only 11% of AR‐V7–positive patients, compared to 30% in AR‐V7–negative cases, underscoring the limited response in this subgroup.^11^ Importantly, our findings demonstrate that not only AR‐V7 but also *AR* amplifications and point mutations negatively impact clinical response across all affected patients. Regardless of initial treatment response, all patients with *AR* alterations ultimately experienced disease progression, as indicated by increasing PSA levels at or shortly after the time of alteration detection. This observation is particularly relevant, as only a subset of our cohort was molecularly profiled before therapy initiation. Our data further show that *AR* alterations frequently co‐occur, with 54% of patients exhibiting multiple *AR* alterations, suggesting a cumulative effect on resistance. This is consistent with Ledet et al., who reported co‐existing *AR* amplification and/or hotspot mutations in approximately 40% of 892 mCRPC patients,^25^ and with Del Re et al., who identified *AR* amplification in combination with AR‐V7 expression in 9 of 43 positive patients, underscoring the frequent co‐occurrence of these resistance mechanisms.^15^

Moreover, the median PFS was significantly shorter in *AR* alteration‐positive patients compared to negative patients (11 vs. 52 months, *p =* 0.001). Although the difference in OS (41 vs. 74 months) did not reach statistical significance (*p =* 0.0619), likely due to sample size limitations and treatment variability, the trend suggests that *AR* alterations may be associated with more aggressive disease phenotypes. Our results align with previous reports linking *AR* alterations to poor outcomes. Romanel et al. found that L702H and T878A mutations were significantly associated with shorter PFS and OS in abiraterone‐treated patients.^26^ Similarly, Conteduca et al. reported these mutations in 8 of 171 ARSi‐treated patients, also correlating with reduced OS.^16^ Del Re et al. observed significantly worse median PFS in AR‐V7‐positive vs. negative patients (PFS: 5.4 vs. 24.3 months).^15^

Our study underscores the importance of continuous *AR* monitoring throughout treatment rather than relying solely on baseline assessments. *AR* alterations were not always present at treatment initiation but emerged in some patients as the disease progressed. Specifically, five patients developed *AR* alterations after starting ARSi therapy, highlighting the dynamic nature of *AR* alterations during treatment. This finding aligns with previous research by Nakazawa et al., which demonstrated that AR‐V7 expression in circulating tumor cells can evolve and even disappear over the course of therapy.^27^

The emergence of *AR* alterations in patients who initially responded to ARSi but later developed resistance suggests a potential role in acquired treatment failure rather than primary resistance. Notably, in one longitudinally monitored case, an *AR* amplification was detected prior to a PSA rise, demonstrating that liquid biopsy can reveal molecular progression earlier than conventional markers and may enable more timely therapeutic interventions.

Our findings also suggest that patients with *AR* alterations may benefit more from taxane‐based chemotherapy than from ARSi therapy. Previous studies, including those by Antonarakis et al., have demonstrated that patients with AR‐V7‐positive metastatic CRPC respond more favorably to chemotherapy compared to ARSi.^8^, ^11^, ^14^, ^28^ Consistent with this, six patients in this study who transitioned to chemotherapy after failing ARSi therapy exhibited superior clinical responses. While this observation reinforces the potential predictive value of *AR* alterations in guiding treatment selection, it is based on a small sample size, and larger studies are needed to validate these findings. This is particularly relevant in light of a contrasting report by Palmberg et al., who described a patient with *AR* amplification showing a superior response to maximal androgen blockade compared to preceding chemotherapy.^29^ Nonetheless, these data contribute to the growing body of evidence suggesting that chemotherapy may be a more effective treatment option for *AR* alteration‐positive patients and highlight the potential role of *AR* biomarker testing in optimizing therapeutic sequencing.

This study has some limitations, including a small and heterogeneous sample size and the absence of standardized treatment protocols. However, these limitations also reflect the real‐world clinical scenario, where treatment decisions are often adapted to individual patient needs rather than following strict protocol‐driven interventions. Instead, this observational study provides pragmatic evidence of the potential relevance of *AR* alterations in the management of mCRPC in real‐world oncology practice.

In recent years, the therapeutic landscape of prostate cancer has evolved significantly, particularly with the earlier introduction of ARSi therapy in hormone‐sensitive stages of the disease. This shift in treatment sequencing has important implications for resistance mechanisms and therapeutic outcomes. Previous studies have reported limited efficacy of sequential ARSi use in advanced prostate cancer, emphasizing the need for biomarker‐driven treatment strategies to optimize patient selection and improve therapeutic outcomes.^6^, ^30^, ^31^, ^32^, ^33^

Our study confirms the importance of longitudinal *AR* monitoring as an important additional tool in clinical practice. Liquid biopsy provides a minimally invasive, patient‐friendly, and effective method for serial monitoring of *AR* status. Unlike tissue biopsies, which are often challenging to obtain in advanced prostate cancer, liquid biopsy enables real‐time assessment of tumor evolution, allowing for earlier detection of treatment resistance and timely adjustments to therapeutic strategies. Moreover, the use of cfDNA and cfRNA in combination with ddPCR is cost‐effective and easily implementable in most laboratories, making it an accessible tool for widespread clinical application. This combination of affordability, accessibility, and real‐time monitoring capabilities highlights the clinical applicability of *AR* alteration screening as a biomarker‐driven tool in CRPC management.

In conclusion, this real‐world observational study demonstrates that monitoring *AR* alterations via liquid biopsy is a promising strategy for guiding treatment decisions in mCRPC. *AR* alterations are associated with reduced ARSi efficacy, shorter PFS, and a trend toward decreased OS. Therefore, patients with *AR* mutations may derive greater benefit from chemotherapy. The dynamic nature of *AR* alterations throughout disease progression underscores the necessity of continuous monitoring to optimize therapeutic strategies. However, future prospective studies with serial sampling before and during ARSi treatment are needed to better characterize the dynamics of *AR* alterations.

Integrating liquid biopsy‐based *AR* testing into routine clinical practice may improve personalized treatment approaches, enabling earlier detection of resistance and more informed therapeutic adjustments. These findings support the potential for *AR* biomarker testing to enhance patient outcomes in mCRPC, particularly as the field moves toward precision medicine in advanced prostate cancer care.

## AUTHOR CONTRIBUTIONS

**Regina Stitz:** Conceptualization; writing – original draft; methodology; validation; visualization; project administration; data curation; formal analysis. **Franz Stoiber:** Investigation; writing – review and editing; project administration; data curation. **Renè Silye:** Supervision; resources; writing – review and editing; project administration. **Elisabeth Rebhan:** Data curation; methodology; writing – review and editing. **Michael Dunzinger:** Investigation; writing – review and editing; resources. **Franz Pühringer:** Writing – review and editing; investigation; methodology. **Ellen Heitzer:** Supervision; writing – original draft; writing – review and editing; methodology; formal analysis. **Cornelia Hauser‐Kronberger:** Writing – review and editing; project administration; supervision.

## CONFLICT OF INTEREST STATEMENT

The authors declare no conflict of interest.

## ETHICS STATEMENT

This study was conducted in accordance with the principles of the Declaration of Helsinki. Ethical approval was obtained from the Ethics Committee of Upper Austria (Approval No. 1271/2021). Written informed consent was obtained from all participants prior to their inclusion in the study. The Ethics Committee raised no objections to the conduct of the study.

## Supporting information


**FIGURE S1.** Individual PSA progression in *AR* alteration positive patients. Panels A–M depict individual PSA trajectories (log_10_ scale) for all *AR* alteration–positive patients, spanning from the initiation of first‐line ARSi therapy to either the end of study observation or patient death. Each panel includes the detection of *AR* status and highlights corresponding treatment interventions. Treatments are represented as ARSi therapy (light blue with various patterns), chemotherapy (pink), radioligand therapy (olive), or no systemic therapy (gray). *AR*, androgen receptor; SNVs*, *AR* p.T878A/S‐F877L + *AR* p.L702H‐W742L/C; ARSi, androgen signaling inhibitor therapy; ADT, androgen deprivation therapy; PSA, prostate‐specific antigen.
**FIGURE S2.** Individual PSA trajectories in *AR* alteration‐negative patients with biochemical, clinical, or radiographic progression. Panels A–I display individual PSA trajectories (log_10_ scale) for nine *AR* alteration–negative patients who experienced tumor progression during the study period. Each trajectory spans from the initiation of first‐line ARSi therapy to either the end of study observation or the patient's death. Each panel includes the *AR* status and highlights relevant therapeutic interventions. Treatments are represented as ARSi therapy (light blue with various patterns), chemotherapy (pink), radioligand therapy (olive), or no systemic therapy (gray). Of the nine patients, two showed no PSA response immediately after the start of ARSi therapy (B and F), while seven initially responded to therapy but later developed tumor progression. AR, androgen receptor; ARSi, androgen receptor signaling inhibitor; ADT, androgen deprivation therapy; PSA, prostate‐specific antigen.
**FIGURE S3.** Individual PSA trajectories in *AR* alteration‐negative patients without biochemical, clinical, or radiographic progression. Panels A–Q display individual PSA trajectories (log_10_ scale) of 17 patients who remained negative for *AR* alterations and exhibited no evidence of biochemical, clinical, or radiographic progression throughout the study period. Each trajectory spans from the initiation of first‐line ARSi therapy to either the end of study observation or the patient's death. Each panel includes the *AR* status and highlights relevant therapeutic interventions. Treatments are represented as ARSi therapy (light blue with various patterns), chemotherapy (pink), radioligand therapy (olive), or no systemic therapy (gray). All patients demonstrated durable treatment response and maintained stable disease throughout the observation period, with consistent *AR* alteration–negative status confirmed across timepoints. AR, androgen receptor; ARSi, androgen receptor signaling inhibitor; ADT, androgen deprivation therapy; PSA, prostate‐specific antigen.

## Data Availability

The data supporting the findings of this study are provided within the article and its supplementary material. Further information is available from the corresponding author upon request.

## References

[1] Siegel RL , Giaquinto AN , Jemal A . Cancer statistics, 2024. CA Cancer J Clin. 2024;74(1):12‐49. doi:10.3322/caac.21820 38230766

[2] Santucci C , Mignozzi S , Malvezzi M , et al. European cancer mortality predictions for the year 2024 with focus on colorectal cancer. Ann Oncol. 2024;35(3):308‐316. doi:10.1016/j.annonc.2023.12.003 38286716

[3] Abou D , Benabdallah N , Jiang W , et al. Prostate cancer theranostics: an overview. Front Oncol. 2020;10:884. 32582550 10.3389/fonc.2020.00884 PMC7290246

[4] Fujita K , Nonomura N . Role of androgen receptor in prostate cancer: a review. World J Mens Health. 2019;37(3):288‐295. doi:10.5534/wjmh.180040 30209899 PMC6704300

[5] Kirby M , Hirst C , Crawford ED . Characterising the castration‐resistant prostate cancer population: a systematic review. Int J Clin Pract. 2011;65(11):1180‐1192. doi:10.1111/j.1742-1241.2011.02799.x 21995694

[6] de Bono J , Mateo J , Fizazi K , et al. Olaparib for metastatic castration‐resistant prostate cancer. N Engl J Med. 2020;382(22):2091‐2102. doi:10.1056/NEJMoa1911440 32343890

[7] de Bono JS , Logothetis CJ , Molina A , et al. Abiraterone and increased survival in metastatic prostate cancer. N Engl J Med. 2011;364(21):1995‐2005. doi:10.1056/NEJMoa1014618 21612468 PMC3471149

[8] Antonarakis ES , Lu C , Luber B , et al. Androgen receptor splice variant 7 and efficacy of Taxane chemotherapy in patients with metastatic castration‐resistant prostate cancer. JAMA Oncol. 2015;1(5):582‐591. doi:10.1001/jamaoncol.2015.1341 26181238 PMC4537351

[9] del Re M , Biasco E , Crucitta S , et al. The detection of androgen receptor splice variant 7 in plasma‐derived Exosomal RNA strongly predicts resistance to hormonal therapy in metastatic prostate cancer patients. Eur Urol. 2017;71(4):680‐687. doi:10.1016/j.eururo.2016.08.012 27733296

[10] Antonarakis ES , Lu C , Luber B , et al. Clinical significance of androgen receptor splice Variant‐7 mRNA detection in circulating tumor cells of men with metastatic castration‐resistant prostate cancer treated with first‐ and second‐line abiraterone and enzalutamide. JCO. 2017;35(19):2149‐2156. doi:10.1200/JCO.2016.70.1961 PMC5493048 28384066

[11] Armstrong AJ , Luo J , Nanus DM , et al. Prospective multicenter study of circulating tumor cell AR‐V7 and Taxane versus hormonal treatment outcomes in metastatic castration‐resistant prostate cancer. JCO Precis Oncol. 2020;4:4. doi:10.1200/PO.20.00200 PMC7608579 33154984

[12] Chandrasekar T , Yang JC , Gao AC , Evans CP . Targeting molecular resistance in castration‐resistant prostate cancer. BMC Med. 2015;13(1):206. doi:10.1186/s12916-015-0457-6 26329698 PMC4556222

[13] Antonarakis ES , Lu C , Wang H , et al. AR‐V7 and resistance to enzalutamide and abiraterone in prostate cancer. N Engl J Med. 2014;371(11):1028‐1038. doi:10.1056/NEJMoa1315815 25184630 PMC4201502

[14] Armstrong AJ , Luo J , Anand M , et al. AR‐V7 and prediction of benefit with taxane therapy: final analysis of PROPHECY. JCO. 2020;38(6_suppl):184. doi:10.1200/JCO2020.38.6_suppl.184

[15] del Re M , Conteduca V , Crucitta S , et al. Androgen receptor gain in circulating free DNA and splicing variant 7 in exosomes predict clinical outcome in CRPC patients treated with abiraterone and enzalutamide. Prostate Cancer Prostatic Dis. 2021;24(2):524‐531. doi:10.1038/s41391-020-00309-w 33500577 PMC8134038

[16] Conteduca V , Wetterskog D , Sharabiani MTA , et al. Androgen receptor gene status in plasma DNA associates with worse outcome on enzalutamide or abiraterone for castration‐resistant prostate cancer: a multi‐institution correlative biomarker study. Ann Oncol. 2017;28(7):1508‐1516. doi:10.1093/annonc/mdx155 28472366 PMC5834043

[17] Danila DC , Samoila A , Patel C , et al. Clinical validity of detecting circulating tumor cells by AdnaTest assay compared with direct detection of tumor mRNA in stabilized whole blood, as a biomarker predicting overall survival for metastatic castration‐resistant prostate cancer patients. Cancer J. 2016;22(5):315‐320. doi:10.1097/PPO.0000000000000220 27749322 PMC5108569

[18] Cattrini C , Rubagotti A , Zinoli L , et al. Role of circulating tumor cells (CTC), androgen receptor full length (AR‐FL) and androgen receptor splice variant 7 (AR‐V7) in a prospective cohort of castration‐resistant metastatic prostate cancer patients. Cancers (Basel). 2019;11(9):1365. doi:10.3390/cancers11091365 31540293 PMC6770005

[19] Khan T , Becker TM , Scott KF , et al. Prognostic and predictive value of liquid biopsy‐derived androgen receptor variant 7 (AR‐V7) in prostate cancer: a systematic review and meta‐analysis. Front Oncol. 2022;12:868031. doi:10.3389/fonc.2022.868031 35372002 PMC8971301

[20] Stitz R , Stoiber F , Silye R , et al. Clinical implementation of a noninvasive, multi‐analyte droplet digital PCR test to screen for androgen receptor alterations. J Mol Diagn. 2024;26(6):467‐478. doi:10.1016/j.jmoldx.2024.02.009 38522838 PMC12178387

[21] Attard G , Reid AHM , Auchus RJ , et al. Clinical and biochemical consequences of CYP17A1 inhibition with abiraterone given with and without exogenous glucocorticoids in castrate men with advanced prostate cancer. J Clin Endocrinol Metab. 2012;97(2):507‐516. doi:10.1210/jc.2011-2189 22170708

[22] Kohli M , Ho Y , Hillman DW , et al. Androgen receptor variant AR‐V9 is Coexpressed with AR‐V7 in prostate cancer metastases and predicts abiraterone resistance. Clin Cancer Res. 2017;23(16):4704‐4715. doi:10.1158/1078-0432.CCR-17-0017 28473535 PMC5644285

[23] Prekovic S , van Royen ME , Voet ARD , et al. The effect of F877L and T878A mutations on androgen receptor response to enzalutamide. Mol Cancer Ther. 2016;15(7):1702‐1712. doi:10.1158/1535-7163.MCT-15-0892 27196756

[24] Rathkopf DE , Smith MR , Ryan CJ , et al. Androgen receptor mutations in patients with castration‐resistant prostate cancer treated with apalutamide. Ann Oncol. 2017;28(9):2264‐2271. doi:10.1093/annonc/mdx283 28633425 PMC5834046

[25] Ledet EM , Lilly MB , Sonpavde G , et al. Comprehensive analysis of AR alterations in circulating tumor DNA from patients with advanced prostate cancer. Oncologist. 2020;25(4):327‐333. doi:10.1634/theoncologist.2019-0115 32297439 PMC7160408

[26] Romanel A , Gasi Tandefelt D , Conteduca V , et al. Plasma AR and abiraterone‐resistant prostate cancer. Sci Transl Med. 2015;7(312):312re10. doi:10.1126/scitranslmed.aac9511 PMC6112410 26537258

[27] Nakazawa M , Lu C , Chen Y , et al. Serial blood‐based analysis of AR‐V7 in men with advanced prostate cancer. Ann Oncol. 2015;26(9):1859‐1865. doi:10.1093/annonc/mdv282 26117829 PMC4551160

[28] Onstenk W , Sieuwerts AM , Kraan J , et al. Efficacy of Cabazitaxel in castration‐resistant prostate cancer is independent of the presence of AR‐V7 in circulating tumor cells. Eur Urol. 2015;68(6):939‐945. doi:10.1016/j.eururo.2015.07.007 26188394

[29] Palmberg C , Koivisto P , Hyytinen E , et al. Androgen receptor gene amplification in a recurrent prostate cancer after monotherapy with the nonsteroidal potent antiandrogen Casodex (bicalutamide) with a subsequent favorable response to maximal androgen blockade. Eur Urol. 1997;31(2):216‐219. doi:10.1159/000474453 9076469

[30] de Wit R , Bono J , Sternberg CN , et al. Cabazitaxel versus abiraterone or enzalutamide in metastatic prostate cancer. N Engl J Med. 2019;381(26):2506‐2518. doi:10.1056/NEJMoa1911206 31566937

[31] Hussain M , Mateo J , Fizazi K , et al. Survival with olaparib in metastatic castration‐resistant prostate cancer. N Engl J Med. 2020;383(24):2345‐2357. doi:10.1056/NEJMoa2022485 32955174

[32] Attard G , Borre M , Gurney H , et al. Abiraterone alone or in combination with enzalutamide in metastatic castration‐resistant prostate cancer with rising prostate‐specific antigen during enzalutamide treatment. JCO. 2018;36(25):2639‐2646. doi:10.1200/JCO.2018.77.9827 PMC6118405 30028657

[33] Hamid AA , Sayegh N , Tombal B , et al. Metastatic hormone‐sensitive prostate cancer: toward an era of adaptive and personalized treatment. Am Soc Clin Oncol Educ Book. 2023;43:e390166. doi:10.1200/EDBK_390166 37220335

