# Supplementary material for: Clinical impact of real‐time androgen receptor alteration monitoring on metastatic castration‐resistant prostate cancer treatment in real‐world settings
Source: Int J Cancer. 2025 Jun 25;157(10):2124–34. doi: 10.1002/ijc.70019 (PMC12439075; doi:10.1002/ijc.70019)
Supplement: Supplementary file 1 — FIGURE S1. Individual PSA progression in AR alteration positive patients. Panels A–M depict individual PSA trajectories (log10 scale) for all AR alteration–positive patients, spanning from the initiation of first‐line ARSi therapy to either the end of study observation or patient death. Each panel includes the detection of AR status and highlights corresponding treatment interventions. Treatments are represented as ARSi therapy (light blue with various patterns), chemotherapy (pink), radioligand therapy (olive), or no systemic therapy (gray). AR, androgen receptor; SNVs*, AR p.T878A/S‐F877L + AR p.L702H‐W742L/C; ARSi, androgen signaling inhibitor therapy; ADT, androgen deprivation therapy; PSA, prostate‐specific antigen. FIGURE S2. Individual PSA trajectories in AR alteration‐negative patients with biochemical, clinical, or radiographic progression. Panels A–I display individual PSA trajectories (log10 scale) for nine AR alteration–negative patients who experienced tumor progression during the study period. Each trajectory spans from the initiation of first‐line ARSi therapy to either the end of study observation or the patient's death. Each panel includes the AR status and highlights relevant therapeutic interventions. Treatments are represented as ARSi therapy (light blue with various patterns), chemotherapy (pink), radioligand therapy (olive), or no systemic therapy (gray). Of the nine patients, two showed no PSA response immediately after the start of ARSi therapy (B and F), while seven initially responded to therapy but later developed tumor progression. AR, androgen receptor; ARSi, androgen receptor signaling inhibitor; ADT, androgen deprivation therapy; PSA, prostate‐specific antigen. FIGURE S3. Individual PSA trajectories in AR alteration‐negative patients without biochemical, clinical, or radiographic progression. Panels A–Q display individual PSA trajectories (log10 scale) of 17 patients who remained negative for AR alterations and exhibited no evidence of [file IJC-157-2124-s001.pdf]

Clinical Impact of Real-Time Androgen Receptor Alteration Monitoring on Metastatic Castration-Resistant Prostate Cancer Treatment in Real-World Settings

Regina Stitz, Franz Stoiber, Renè Silye, Elisabeth Rebhan, Michael Dunzinger, Franz Pühringer, Ellen Heitzer, Cornelia Hauser-Kronberger

Content

Supplemental Figure 1. Individual PSA progression in *AR* alteration positive patients........... 2

Supplemental Figure 2. Individual PSA trajectories in *AR* alteration-negative patients with biochemical, clinical, or radiographic progression.. 3

Supplemental Figure 3. Individual PSA trajectories in *AR* alteration-negative patients without biochemical, clinical, or radiographic progression.. 4

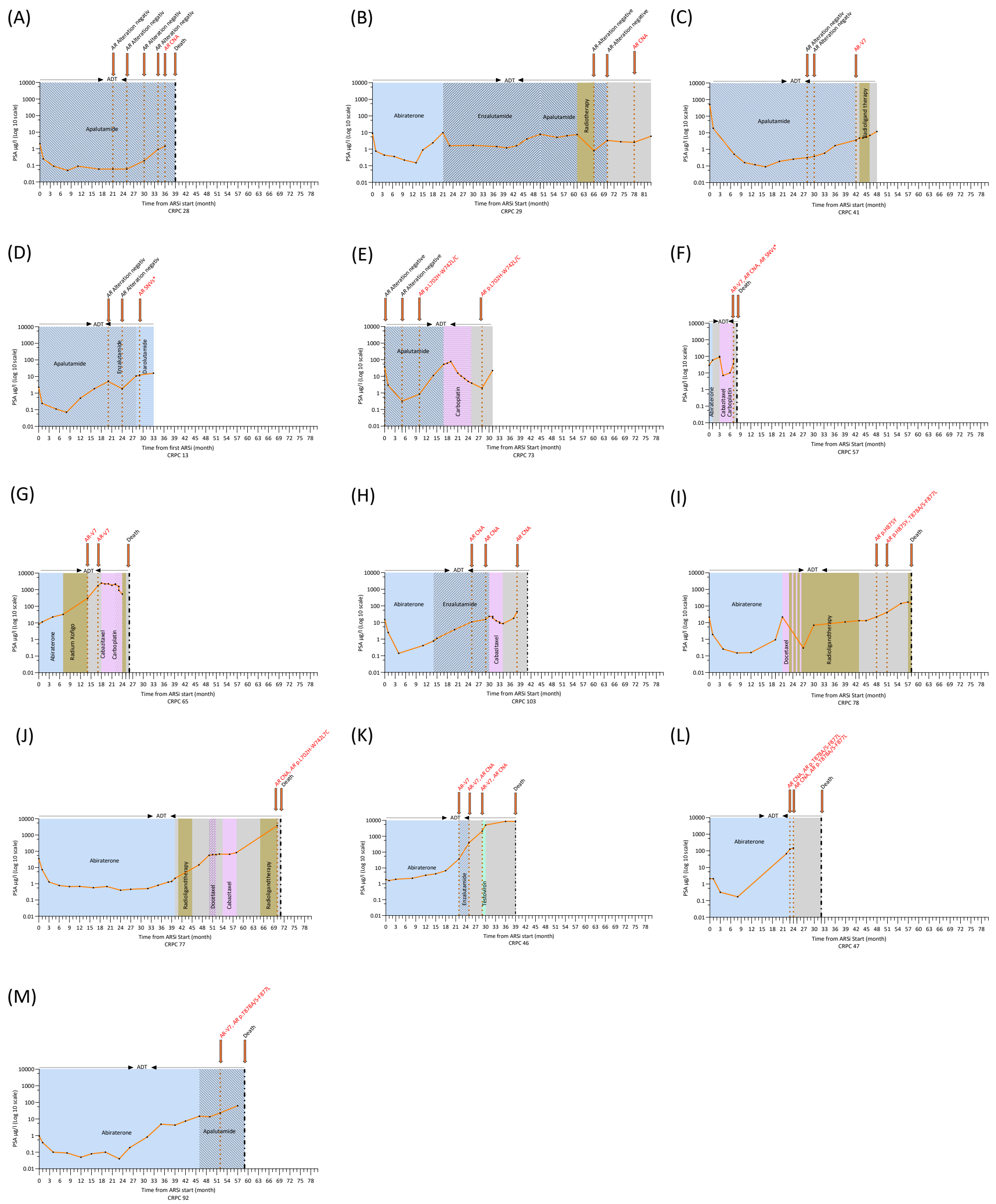

Supplemental Figure 1. Individual PSA progression in *AR* alteration positive patients
Panels A–M depict individual PSA trajectories ($\log_{10}$ scale) for all *AR* alteration–positive patients, spanning from the initiation of first-line ARSi therapy to either the end of study observation or patient death. Each panel includes the detection of *AR* status and highlights corresponding treatment interventions. Treatments are represented as ARSi therapy (light blue with various patterns), chemotherapy (pink), radioligand therapy (olive), or no systemic therapy (gray). Abbreviations: *AR*, androgen receptor; SNVs*, *AR* p.T878A/S-F877L+*AR* p.L702H-W742L/C; ARSi, androgen signaling inhibitor therapy; ADT, androgen deprivation therapy; PSA, prostate-specific antigen.

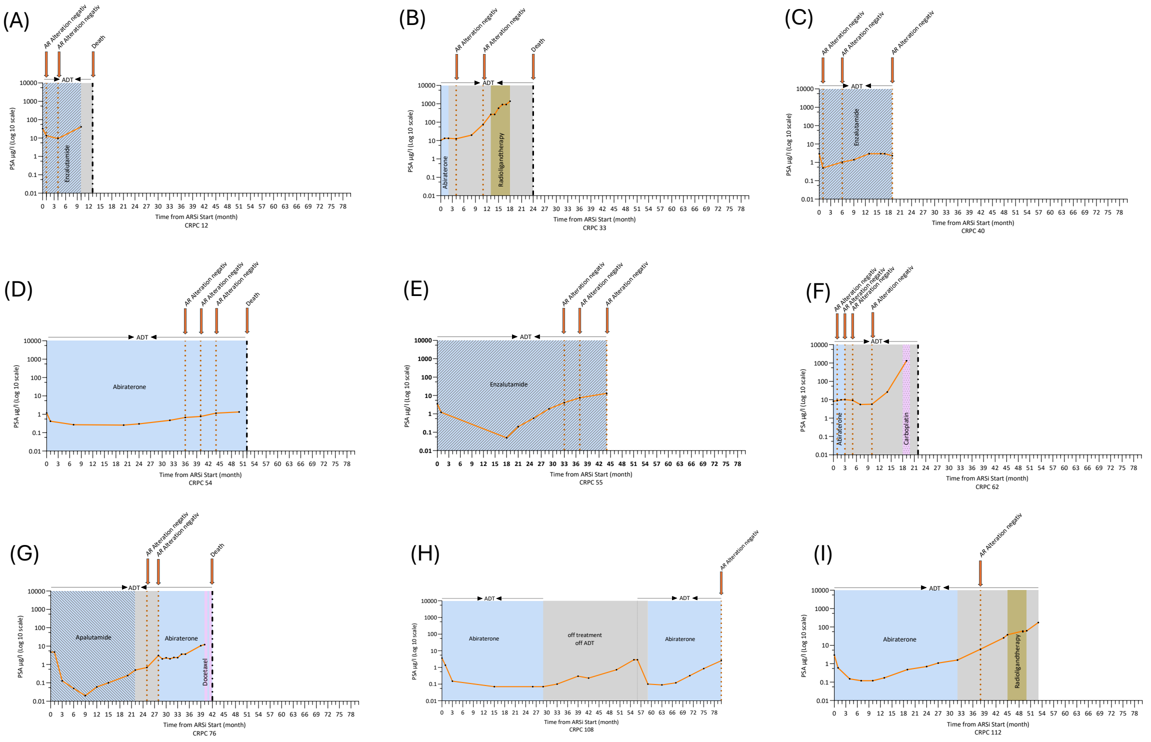

Supplemental Figure 2. Individual PSA trajectories in *AR* alteration-negative patients with biochemical, clinical, or radiographic progression

Panels A–I display individual PSA trajectories ($\log_{10}$ scale) for nine *AR* alteration–negative patients who experienced tumor progression during the study period. Each trajectory spans from the initiation of first-line ARSi therapy to either the end of study observation or the patient's death. Each panel includes the *AR* status and highlights relevant therapeutic interventions. Treatments are represented as ARSi therapy (light blue with various patterns), chemotherapy (pink), radioligand therapy (olive), or no systemic therapy (gray). Of the nine patients, two showed no PSA response immediately after the start of ARSi therapy (Figures B and F), while seven initially responded to therapy but later developed tumor progression. Abbreviations: AR, androgen receptor; ARSi, androgen receptor signaling inhibitor; ADT, androgen deprivation therapy; PSA, prostate-specific antigen.

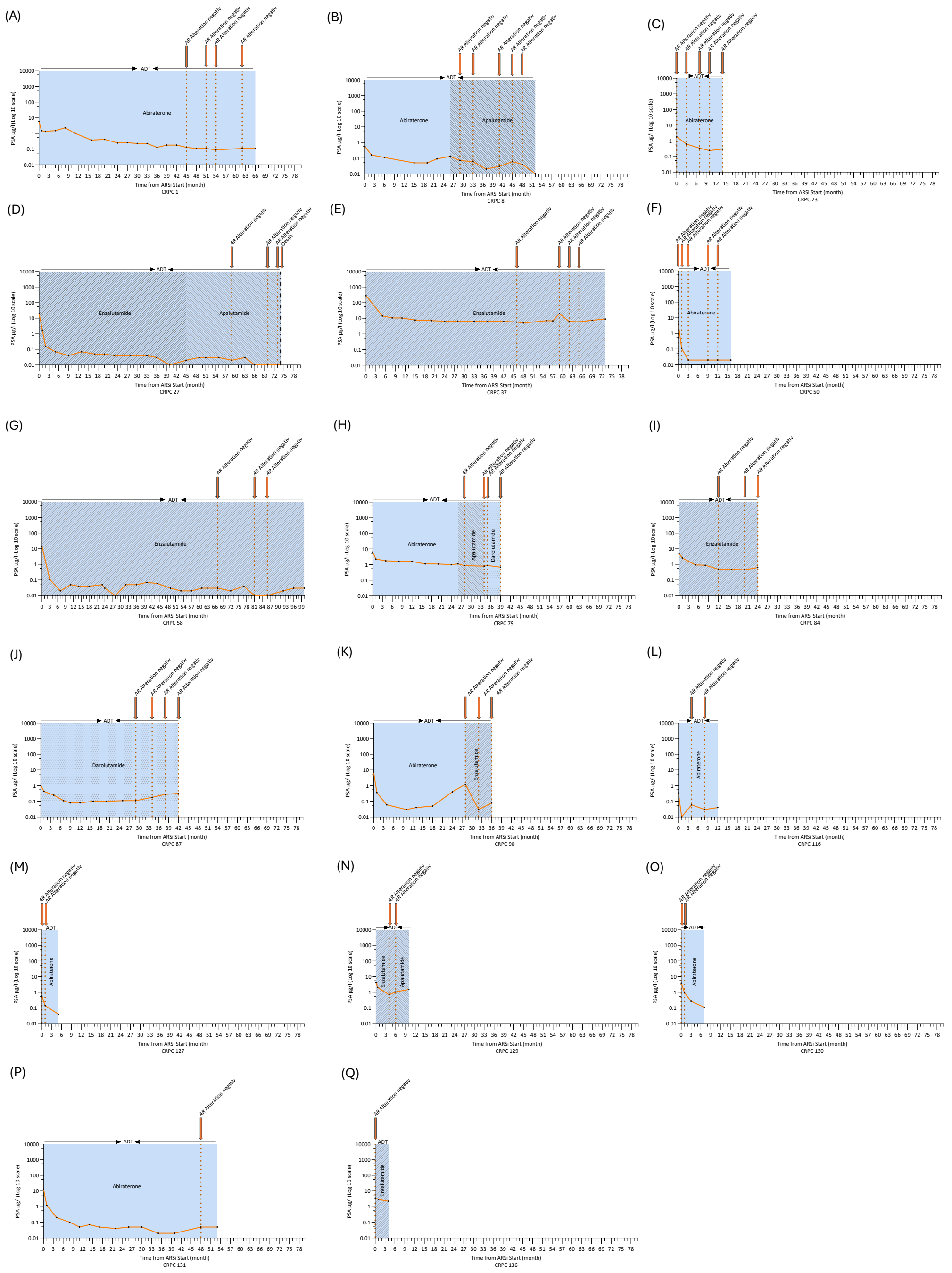

Supplemental Figure 3. Individual PSA trajectories in *AR* alteration-negative patients without biochemical, clinical, or radiographic progression

Panels A–Q display individual PSA trajectories ($\log_{10}$ scale) of 17 patients who remained negative for *AR* alterations and exhibited no evidence of biochemical, clinical, or radiographic progression throughout the study period. Each trajectory spans from the initiation of first-line ARSi therapy to either the end of study observation or the patient's death. Each panel includes the *AR* status and highlights relevant therapeutic interventions. Treatments are represented as ARSi therapy (light blue with various patterns), chemotherapy (pink), radioligand therapy (olive), or no systemic therapy (gray). All patients demonstrated durable treatment response and maintained stable disease throughout the observation period, with consistent *AR* alteration–negative status confirmed across timepoints.

Abbreviations: AR, androgen receptor; ARSi, androgen receptor signaling inhibitor; ADT, androgen deprivation therapy; PSA, prostate-specific antigen.
